# Noninvasive Measurements of Breast Cancer-Related Lymphedema

**DOI:** 10.7759/cureus.19813

**Published:** 2021-11-22

**Authors:** Harvey N Mayrovitz

**Affiliations:** 1 Medical Education, Nova Southeastern University Dr. Kiran C. Patel College of Allopathic Medicine, Fort Lauderdale, USA

**Keywords:** edema measurement, water displacement, limb volumes, impedance, measuring lymphedema, tissue dielectric constant, breast cancer

## Abstract

Breast cancer-related lymphedema (BCRL) presents as swelling in the arm, hand, trunk, or breast at varying times after completion of breast cancer treatment. Its reported incidence varies widely in part due to its dependence on the type and extent of the treatment, co-present pre-treatment risk factors, and the criteria used to define its presence. Central to this issue is the quantitative measures that are variously used to specify lymphedema thresholds for its detection and tracking over time and during treatment. The goal of this paper is to discuss these issues and the methods available for the non-invasive quantitative assessment of BCRL. Operational principles and advantages and limitations of the various methods and their clinical history of use and effectiveness are discussed.

## Introduction and background

Breast cancer-related lymphedema (BCRL) has a variable reported incidence that depends on the objective criteria used and, on the type and extent of a patient’s treatment [1,2]. When BCRL occurs, it manifests itself as increased fluid anywhere in the at-risk arm or thorax, or other areas [3]. Most readily assessable noninvasive methods that quantify its presence and extent are restricted to those that measure arm size change or the difference between at-risk arm and contralateral arm. These methods include manual and automated circumference and volume measurements, arm fluid changes assessed by electrical impedance, and volume changes assessed by water displacement measurements (WDM) [4-6]. There is also a method that is fully portable that may be used to assess localized lymphedema at any anatomical site via tissue dielectric constant measurements [7]. Knowledge of operational principles and advantages and limitations of these various methods and their clinical history of use and effectiveness are important considerations that impact both clinical and research decisions in choosing measurement approaches. 

## Review

The goal of this paper is to discuss and elucidate the measurement methods and clarify their potential usefulness with a focus on readily available measurement methods for the non-invasive quantitative assessment of BCRL.

Arm parameters based on manual circumference measurements

The circumference of the non-compressed and relaxed arm is measured with a tape measure with the tape measure optimally pulled with a constant tension using a spring-loaded calibrated tape measure [4]. Such measurements are done at multiple arm locations. Practical measurement issues include the number of circumferential measurements to be made and their longitudinal separation, the volume calculation model to be used to incorporate the measured circumferences, and how the volumes or circumferences are optimally used to assess initial lymphedema status and to track its change due to either time or therapy.

These measurement issues have been investigated in healthy and lymphedematous arms. Casley-Smith was among the first to systematically study these issues [8]. An approach was to calculate segment volumes (VS) between two circumferences (c1,2) separated by a distance L using a truncated cone model as VS = L/(12$\pi$) x [c_1_^2^+c_1_c_2_+c_2_^2^). The formula was used in 150 unilateral BCRL patients with circumferences measured at mid-hand, the narrowest part of the wrist, and at 10 cm intervals starting from the middle fingertip. By summing segment volumes, arm volumes of interest were determined. Percentage edema volume (%EV) was calculated as the volume difference between affected and contralateral arms divided by contralateral arm volume. The %EV determined this way was compared to edema volumes determined via water displacement (%EVW), considered as the gold standard. A high correlation between methods was reported (r=0.925) with a regression equation %EV=1.096 %EVW + 0.007. Another study by Ward et al. suggested that rather than using a truncated-cone model, a summation-of-disks model might be better [9].

Measurements in 15 BCRL patients compared segment lengths of 10-cm vs. about 4-cm in which the 4-cm start was the most distal portion of the wrist and the end at about 45 cm [4]. Calculated %EV was similar for both, but shorter segments had a statistically greater %EV that was most evident at greater arm volumes and had better accuracy. The circumference-volume method was also compared to WDM on 14-women with unilateral BCRL using 4-cm segment lengths [10]. Small differences in arm volumes were reported depending on the method, but inter-arm differentials were highly correlated (r=0.79). A subsequent reliability and validity study compared the circumference-volume method with WDM on 66 women in which 19 had unilateral BCRL [11]. Measured arm length was standardized to start at the wrist (mid-ulnar styloid) and extend to 65% of the distance from the olecranon (elbow) to the acromion (shoulder tip). Circumferential measurements were made at specified anatomical sites or standardized distances, with four segments used to calculate the volume and compared to WDM. Results indicated a good correlation between methods but, based on limits of agreement, the methods could not reliably be interchanged since calculated volumes were up to 5% greater than WDM values. Based on reliability analyses they concluded that the minimal detectible change in volume that could be used as a clinical threshold representing a real change due to time or treatment was 150 ml.

Arm volumes from automated circumference measurements

**Figure 1:**
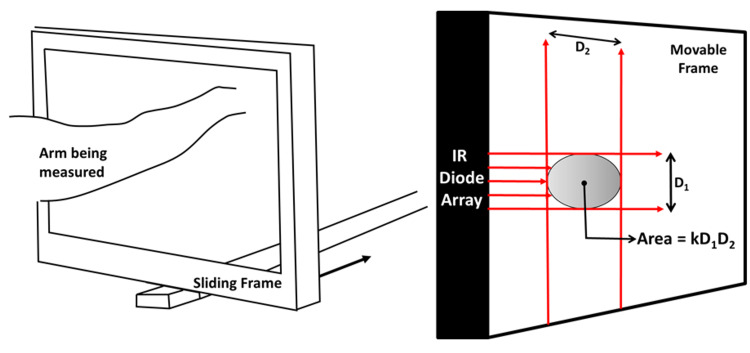
Basic principle and elements of automated optoelectronic arm measurements

Automation of manual circumference measurements emerged with the arrival of a device commercially known as the Perometer [5]. Its basic operating principle is illustrated in Figure 1. A sliding frame with embedded infrared (IR) light sources scans the arm and the “shadow” dimensions D1 and D2 are detected and used to calculate cross-sectional areas as a constant (k) multiplied by D1 and D2. Segment volumes are determined similarly to the manual method with segment volumes summed to produce the arm volume of interest. This method has the advantage of rapidly estimating cross-sectional areas using as low as 0.5 cm segment lengths and an automatic calculation of arm volumes. Disadvantages include device set-up, space requirements, patient positioning, service maintenance, and initial cost as compared to manual methods. 

Initial evaluations on 17 lymphedematous arms indicated an arm volume 6.8 ± 4.3% greater than manual tape measure values [5]. Subsequent tests on 37 lymphedematous arms compared automated volumes vs. tape measure (5-cm intervals) vs. WDM [12]. The reproducibility of each method was assessed as satisfactory with inter- and intra-class correlation coefficients ranging from 0.937 to 0.997. Repeated measurements by the same rater (intra-rater variation) yielded volume percentage differences of 1.5 ± 1.4% for the Perometer, 2.9 ± 2.9% for WDM, and 3.2 ± 4.6% for the tape measure method. Using perometry to pre-operatively screen 1028 women with unilateral BCRL emphasized the importance of pre-surgical volumes [13].

BCRL thresholds based on arm differentials or changes

Beyond clinical assessments and patient symptoms, various quantitative parameters have been developed to help define BCRL's presence in its early subclinical stage. Parameters based on arm metrics were the earliest and remain in use now, but other methods such as bioimpedance, tissue dielectric constant measurements are now also available as discussed subsequently.

Most BCRL cases are unilateral, so it is common to compare at-risk arms to contralateral arms to inter-arm differentials. Arm circumferences are measured bilaterally at corresponding anatomical sites and inter-arm circumferences or inter-arm volumes are compared. Pioneering work showed that untreated BCRL progresses in volume and grade rapidly at first and slowly thereafter [8]. Untreated BCRL quantified as inter-limb volume ratios (VR) increased by 40.6% in the first year, 12.4% from year one to five, and 4.22% from five to 30 years [14]. Problems of BCRL measurements and accurate BCRL incidence assessments were raised by early investigators and parameter values that best reflect BCRL presence was studied by comparing three lymphedema thresholds based on arm metric measurements [1,15]. Arm circumferences were tape-measured at 4-cm intervals and also measured by perometry before breast surgery and at six and 12-months post-surgery in 110 breast cancer survivors. Incidence was assessed in three ways based on at-risk arms vs. contralateral arms; 2-cm circumference change at any site, 200 ml volume change, and 10% volume change. These thresholds yielded very different one-year incidence rates of 46%, 24%, and 8%, respectively.

Further study on 236 patients followed for up to five years, indicated the 2-cm difference predicted a 94% incidence whereas the 10% volume difference criterion predicted a 45% incidence [16]. More recently, 1100 women with breast cancer (BC) who had axillary lymph node dissection (ALND) were followed for up to five years [17]. The BCRL thresholds used in this multicenter study were a relative arm volume increase of ≥10% (Perometer determined) and a lymphedema index (L-Dex) value greater than 10 based on bioimpedance spectroscopy (BIS). By 24 months, 22.8% had BCRL based on volume but 45.6% had it based on L-Dex [17,18]. Early detection of relative volume increases between 5% to 10 % were strong predictors of BCRL occurring by 36 months. Analyses of the primary study data indicated a median time to develop BCRL was 11.3-months. For women followed up to five years (n=156), 31.9% had BCRL as assessed by volume whereas 77.2% had BCRL according to the 200 ml threshold [19].

BCRL assessments based on water displacement methods

Water displacement measurements (WDM) are considered by many to be the “gold standard” for volume measurements [11]. Insertion of the arm into a water-filled volumeter causes a water volume equal to the inserted arm volume to be displaced and captured as overflow. Although accurate, the method is time-consuming and messy and depends on patient mobility to implement, and is not routinely used in clinics. However, it can provide comparisons against which other methods may be assessed and provide reference values against which BCRL thresholds are developed. Absolute arm volume thresholds are most useful if pre-surgery values are not available.

Water displacement BCRL thresholds

Using WDM, arm volumes were measured in 112 women (50.6 ± 18.2) years with a BMI of 24.5 ± 3.9 Kg/m2 [20]. Most were right-handed (n=100) with right arm volumes about 3% greater than the left. Such handedness differences should be taken into account. Prediction equations extrapolating back to what an arm volume would have been before BCRL may be applied based on normative values. For right-handed women, right arm volume (RAV) in terms of left arm volume (LAV) can be expressed as RAV=0.979 LAV+96.66 ml. Contrastingly, for LAV of right-handed women, the relationship is LAV=0.991 RAV-33.3 ml. In each case, a 95% confidence interval was about 148 ml. It was suggested that the equations, together with upper confidence limits, be used for thresholds.

As an example, consider a right-handed woman with a left-arm at-risk who has a measured RAV of 3000 ml. Her normal (predicted) LAV is 2940 ml to which is added 148 ml resulting in a 2 standard deviations (2SD) threshold of 3088 ml and an inter-arm ratio of 1.029. Contrastingly, if the right arm were at-risk with the left arm measured at 3000 ml, the predicted threshold is 3182 ml with an inter-arm ratio of 1.06.

Inter-arm differentials of 200 ml, determined by WDM, were used as a threshold defining sustained BCRL in 85 women 24 months post-surgery [21]. Accordingly, 19 (22.4%) had BCRL at 24 months. Contrastingly, based on two inter-arm circumference measurements made at six months post-surgery differing by ≥ 2 cm, the calculated probability of sustained BCRL at 24 months was 60%. 

BCRL assessments with bioimpedance spectroscopy

The term bioimpedance spectroscopy (BIS) refers to measuring electrical impedance at multiple frequencies. This method is widely used but it has been argued that it is not a proper substitute for volumes or, for localized assessments of limb lymphedema and is also not applicable to other body areas [9]. Contrastingly, it has been argued that it should be adopted as a gold standard [22]. In some quarters it has become common to use a surrogate parameter called the L-Dex [23].

**Figure 2:**
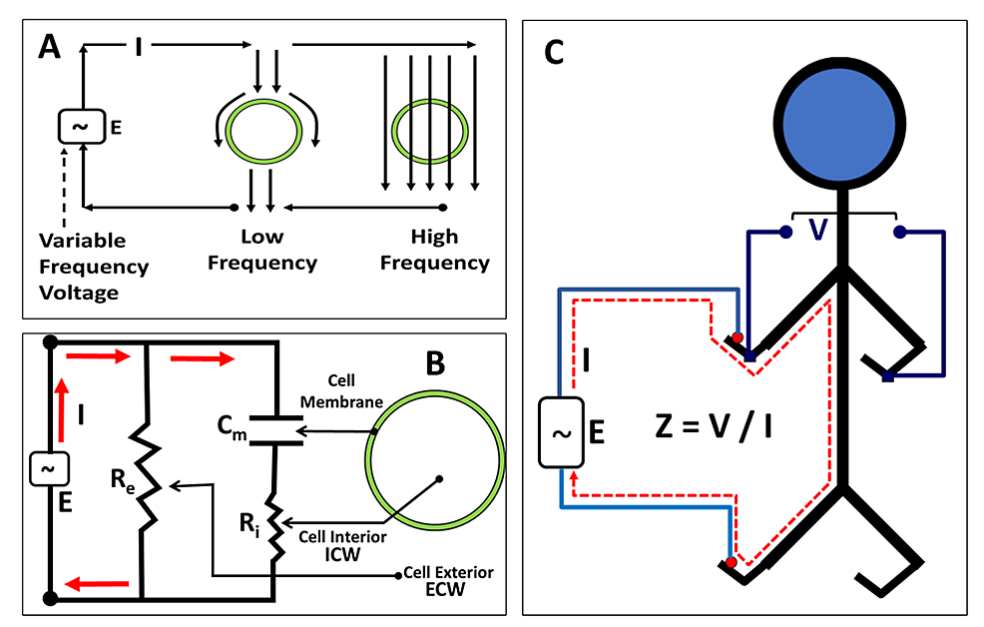
Basic principle and elements of bioimpedance spectroscopy measurements

There is some controversy about whether this parameter and its threshold for BCRL are adequate [24]. Its operational principles are illustrated in Figure 2. Application of a sinusoidal varying voltage causes a time-varying current that depends on the frequency and the current’s pathway (Figure 2 A). At low frequencies, cell membranes pass little or no current due to the membrane’s high electrical capacitance whereas at high frequencies current passes through the cell. As a consequence, the composite pathway may be represented by an equivalent electrical circuit (Figure 2 B). The quantities Re and Ri represent external and internal electrical resistances. These resistance values are inversely related to the amounts of extracellular water (ECW) and intracellular water (ICW) through which the currents flow. Because of the low electrical resistance of body fluids relative to other body components such as fat and connective tissue, the overall measured impedance (Z), which is the ratio of the voltage difference (V) to current (I), is strongly dependent on fluid content. A schematized version of arm impedance is shown in Figure 2 C.

In Figure 2 C, the applied voltage causes an exciting current to flow (dashed lines). Right arm impedance is calculated as V/I where V is the voltage difference between right and left hands. In practice, components attributable to ICW and ECW can be separated. In assessing BCRL the concept is that excess arm fluid causes the affected arm to have a reduced impedance vs. a pre-surgery baseline or vs. the contralateral arm. Devices are available for such measurements (Impedimed Ltd., Brisbane, Australia). One version (model SFB7) uses 256 frequencies that range between 3 KHz to 5 KHz (low frequency) to 1000 KHz (high frequency). The use of multiple frequencies allows extrapolations to evaluate theoretical zero and infinite frequencies based on Cole-Cole plots that describe resistance vs. reactance as a function of frequency [25]. These yield estimates of ECW and ICW.

To determine changes only in ECW, which is the dominant fluid change compartment associated with lymphedema, the multiple frequencies are much less important and a single frequency of less than 30 KHz may be sufficient [26]. An excellent correlation between single frequency and BIS-determined values was obtained for frequencies ≤ 50 KHz [27]. However, whether using single or multiple frequencies, such measurements may not capture the full lymphedema picture because it fails to include the contribution of bound water [28]. Some BIS technology is now incorporated in a system allowing BIS measurements while standing. 

BCRL thresholds based on BIS

Impedance measurements have been used for many years with applications ranging from studying peripheral vasculature to cardiac assessments with impedance cardiography. However, its early description in the assessment of lymphedema can be traced to the early to mid-1990s [29]. The BCRL thresholds were originally based on inter-limb impedance ratios obtained in healthy persons (dominant/non-dominant) arms, with thresholds defined as inter-arm impedance ratios (contralateral/at-risk) exceeding a mean ratio + 3SD as determined in 60 healthy women [30]. The mean and standard deviation (SD) of this ratio was 0.964 ± 0.034 that led to a threshold of 1.066. Strictly speaking, this threshold applies to detecting BCRL in women in whom their dominant arm was at-risk. An adjustment to this threshold ratio made for women whose at-risk arm was their non-dominant arm was reported as 1.139 [31]. The use of the 3SD threshold is arbitrary but represents a conservative estimate that yields a better sensitivity. This threshold was refined from measurements in 172 healthy women in which dominant/non-dominant impedance ratios were 0.986 ± 0.040 yielding a threshold of 1.106. 

BCRL assessments based on tissue dielectric constant

**Figure 3:**
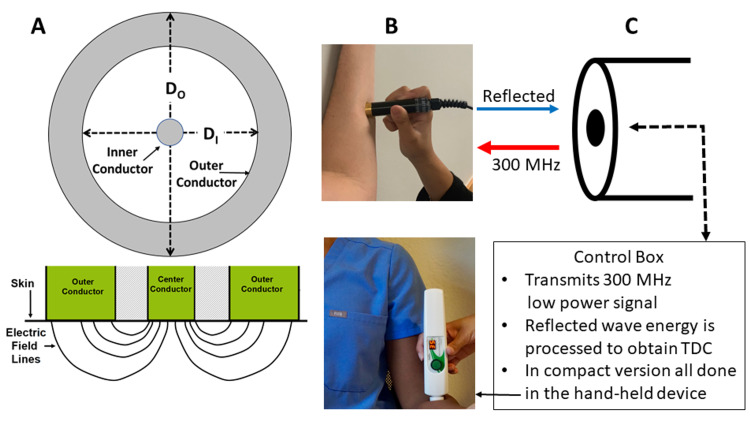
Basic principle and elements of tissue dielectric constant measurements

The term tissue dielectric constant (TDC) was coined in 2007 to represent the value of the relative permittivity of skin-to-fat tissue measured in vivo using the open-ended coaxial line method [7, 32]. To assess edema or lymphedema, its use is based on the fact that its value strongly depends on tissue water content [33]. In contrast to lymphedema assessment methods useful only for limbs, TDC measurements are localized and usable to measure skin water or skin-to-fat water at most anatomical sites including breast and trunk [34-37]. From a physical perspective, tissue permittivity may be thought of as the electric flux density produced when tissue experiences an applied electric field. In this form, permittivity or TDC is the ratio of flux density produced to the electric field causing it. The TDC is measured by touching the skin with a probe that has concentric inner and outer electrodes (conductors) that functions as an open-ended coaxial line (Figure 3 A-top). The probe inserts a 300 MHz electric field from a battery-operated control box or within the probe itself for a compact version (Figure 3 B-bottom). One of the multiprobe types is shown in Figure 3 B-top. For both, a time-varying electric field penetrates the tissue (Figure 3 A-bottom). For a given frequency, the depth of penetration depends on the probe’s radial dimensions with larger diameter probes penetrating deeper [38,39]. Some incident electromagnetic energy is reflected (Figure 3 C) and from an analysis of this component, TDC is determined via algorithms based on the physics of the process [32].

Devices are available (Delfin Technologies, Kuopio, Finland) that provide probes for effective measurement depths between 0.5 mm and 5.0 mm (multiprobe system) or fixed depths (compact versions). In some tissues, TDC values depend on measurement depth because of depth-dependent tissue heterogeneity [40,41]. Increased fat with increasing depth tends to lower the TDC value due to the low water content of fat [42]. Variations in TDC values are also expected based on sex, age, body habitus, and at different anatomical sites along the arm [40,42-44]. These normal biological variations do not importantly impact TDC use as a lymphedema assessment method because of various normalization processes. For unilateral BCRL, inter-arm or inter-trunk, or inter-breast ratios are used with the added advantage that specific localized targets can be tracked. 

BCRL thresholds based on TDC

The TDC measurements to a depth of 2.5 mm in 30 healthy pre- and 30 post-menopausal women yielded an inter-arm TDC ratio of 1.040 ± 0.040 vs. 1.640 ± 0.300 in 18 patients with BCRL [7]. In that study, no healthy control had an inter-arm ratio as great as 1.200 and no patient had a ratio as low as 1.200. It was suggested that an inter-arm TDC ratio of 1.200 should be a BCRL threshold value. Had they used a threshold based on 3SD greater than the mean, it would have been 1.160. Other work supported the 1.200 threshold ratio [45]. Inter-arm TDC ratios were compared between 60 healthy women and 30 patients with unilateral BCRL [46]. The healthy group’s inter-arm TDC ratio (dominant/non-dominant) was 1.006 ± 0.085 with a 3SD TDC-threshold ratio of 1.26. This was initially used to define arm BCRL and was insignificantly affected by patient body mass index (BMI), age, measurement depth, or hand-dominance [47]. Subsequent inter-side measurements made with a compact-type TDC device in 112 breast cancer survivors without BCRL had at-risk to contralateral side ratios for the forearm, upper arm, and middle lateral thorax of 1.00 ± 0.09, 1.01 ± 0.15, and 1.06 ± 0.10, respectively [37]. These ratios differed from those similarly measured in 78 breast cancer survivors diagnosed with BCRL in whom corresponding inter-side ratios were 1.29 ± 0.36, 1.25 ± 0.41, and 1.07 ± 0.12 [37]. In this study, the time since breast surgery was 8.4 ± 6.7 years with 12.8 ± 8.7 nodes removed. Non-BCRL control patients were 6.9 ± 6.7 years post-surgery with 6.1 ± 7.3 nodes removed. Based on a 3SD threshold for control ratios, an inter-arm BCRL threshold may be calculated as 1.27, 1.46, and 1.36 for the forearm, upper arm, and thorax. A slightly lower thorax-to-thorax inter-side ratio of 1.38 has been reported from measurements on 120 women awaiting breast cancer surgery [48]. A reference range for inter-hand TDC ratios has been determined via measurements in 70 healthy women to be 1.326 [47]. A comparison between BIS and TDC methods in 100 women with BCRL has reported TDC having a greater sensitivity at detecting early lymphedema [49].

Discussion

It has been long recognized that in addition to changes in interstitial fluid content accompanying developing lymphedema there are progressive changes in tissue content, structure, and physical properties. Such changes include increases in arm fat, muscle, and bone as has been demonstrated via dual-energy X-ray absorptiometry in patients with BCRL. It is thus likely that such complex changes that do contribute to arm volume increases will not directly be reflected as decreases in measured arm impedance. These complexities have been investigated using MRI in which excess fat volume was observed in both intra- and inter-muscular compartments in patients with BCRL. An increasing relative amount of fat accumulation would increase measured arm impedance even though arm volume was increasing indicating a potential limitation to BIS tracking in such cases.

Other factors to be considered relevant to BCRL portable or semi-portable measurement techniques are their relative initial cost, continuing supply needs, and operating costs, maintenance needs, ease of use, and difficulties with measurement. Based on such considerations, the relatively non-portable Perometer system (350NT) cost is estimated to be at least $ 33,000 so that it may be most useful for high-volume screening facilities. The use of BIS may also be most useful for high-volume screening and follow-up purposes in which sensitivity to small-to-moderate free and bound water increases are not important. The initial clinical unit cost is about $8000 with a continuing electrode cost per patient. Contrastingly, the hand-held TDC device, costing about $4000 and with no subsequent operating costs, may be most useful for a rapid routine physician or therapist use for initial BCRL detection and follow-up assessments of localized BCRL or, for lymphedema measurements at any anatomical site related to BCRL such as breast or thorax.

## Conclusions

Based on the present findings, including comparisons between BIS and TDC methods in women with BCRL, it is concluded that TDC has a greater sensitivity at detecting early localized lymphedema. This may in part be due to lymphedema that is more superficial. Based on the literature it is likely that TDC measurements can serve individually for localized assessments and tracking and also as complementary to volume assessments as needed. The literature also indicates several practical advantages of TDC beyond its portability and ease of use. These include the fact that it is not contraindicated for use in patients who have pacemakers or metal implants and can be used in patients even if in contact with a metal surface. Both BIS and automated circumference measurement approaches also have utility probably mostly in specialized facilities with high-volume throughput. From the point of view of cost, tape measure procedures with volume conversion and limb volumes via water displacement require the least for equipment, but measurement time and patient acceptance need to be considered. Tape measurements are well accepted by patients but may require extended therapist measurement time. In the case of water displacement measurements, patient positioning and discomfort as with all methods are to be considered. The shorter the required measurement time and the less intrusive to the patient the better tolerated is the measurement process. 
